# Overexpression of matrix metalloproteinase-9 (MMP-9) rescues insulin-mediated impairment in the 5XFAD model of Alzheimer’s disease

**DOI:** 10.1038/s41598-017-00794-5

**Published:** 2017-04-06

**Authors:** Archontia Kaminari, Nikolas Giannakas, Athina Tzinia, Effie C. Tsilibary

**Affiliations:** 1 Institute of Biosciences and Applications, National Center for Scientific Research “Demokritos”, Agia Paraskevi, Athens, 15310 Greece; 2 Division of Human and Animal Physiology, Department of Biology, National and Kapodistrian University of Athens, Athens, Greece

## Abstract

A hallmark of Alzheimer’s disease (AD) is the accumulation of oligomeric amyloid-β (Aβ) peptide, which may be primarily responsible for neuronal dysfunction. Insulin signaling provides a defense mechanism against oligomer-induced neuronal loss. We previously described the neuroprotective role of matrix metalloproteinase 9 (MMP-9) in decreasing the formation of Aβ oligomers. In the present study, we examined the role of MMP-9 on the insulin survival pathway in primary hippocampal cultures and hippocampal cell extracts from 3 month-old wild type, AD (5XFAD), MMP-9-overexpressing (TgMMP-9), and double transgenic mice (5XFAD/TgMMP-9). The data demonstrate that the insulin pathway was compromised in samples from 5XFAD mice, when compared to the wild type and TgMMP-9. This was due to enhanced phosphorylation of IRS1 at Serine 636 (pIRS1-Ser636), which renders IRS1 inactive and prevents insulin-mediated signaling. In 5XFAD/TgMMP-9 samples, the insulin survival pathway was rescued through enhanced activation by phosphorylation of IRS1 at Tyrosine 465 (pIRS1-Tyr465), downstream increased phosphorylation of Akt and GSK-3β, and decreased phosphorylation of JNK kinase. Oligomeric Aβ levels decreased and BDNF levels increased in 5XFAD/TgMMP-9 mice, compared to 5XFAD mice. Our findings indicate that overexpression of MMP-9 rescued insulin survival signaling *in vitro* and in early stages in the 5XFAD model of AD.

## Introduction

Alzheimer’s disease (AD) is the most common neurodegenerative disease and is nowadays turning to slow pandemic, due to its high prevalence of occurrence in the elderly population worldwide^1^. One of the hallmarks of the disease includes the formation of amyloid-β (Aβ) oligomers, which subsequently aggregate into the disease’s characteristic insoluble amyloid plaques^2^. Nevertheless, increasing data indicate that the soluble oligomeric form of Aβ is primarily responsible for cell toxicity and neuronal dysfunction in various experimental models of AD^3, 4^, as well as in AD patients^5^. Aβ oligomers are generated via proteolytic cleavage of the amyloid precursor protein (APP) by β- and γ- secretases^6^. Alternatively, APP is cleaved by α-secretases, shifting the pathway in favor of the production of a soluble Ν-terminal fragment APPα (sAPPα), which has been reported to have neurotrophic properties^7, 8^.

The impact of Aβ oligomers in the brain was reported to be related to defective insulin signaling. Insulin confers neuroprotective properties in the central nervous system (CNS) by playing a vital role in learning and memory formation^9, 10^, whereas impairment of insulin has been associated with cognitive deficits in AD patients and in animal models of AD^11^. Oligomer-mediated insulin resistance occurs due to Aβ binding to the neuronal membrane surface, which results in the removal of the insulin receptors (IRs)^5, 12, 13^ at the synaptic area and eventually to synapse loss.

On the contrary, insulin-mediated signaling provides a physiological defense mechanism against oligomer-induced synapse loss by down-regulating oligomeric binding sites^14^. During normal insulin function, insulin binds to its receptor and activates insulin substrate-1 (IRS1) via phosphorylation at Tyrosine residue 465 (pIRS1-Tyr465). In turn, this phosphorylation promotes the neurotrophic effects of insulin by activating cell survival Akt/PKB kinase (Akt). One mode of action of Akt involves the phosphorylation and subsequent inactivation of Glycogen Synthase Kinase-3β (GSK-3β), an enzyme associated with the hyper-phosphorylation of the Tau protein, another pathological marker of the disease^15, 16^.

In early stages of AD, Aβ were reported to accumulate in post-synaptic areas leading to stimulation of pro-apoptotic pathways due to activation of the c-Jun N-terminal kinase (JNK) pathway; this results in phosphorylation of IRS1 at Serine residue 636 (pIRS1-Ser636), which renders IRS1 inactive and suppresses the stimulatory effect of insulin^17^. In later stages of AD this modification results in severe memory impairment and eventually cell death due to IR removal from the neuronal surface^17, 18^. Hence, stimulation of the insulin signaling pathway is a plausible mechanism of protection against Aβ oligomer-induced synapse loss.

Matrix metalloproteinases (MMPs) belong to a family of zinc bound proteolytic enzymes responsible for the remodeling of the extracellular matrix^19^. Matrix metalloproteinase 9 (MMP-9), along with other matrix metalloproteinases, has been reported to play a role in the configuration of synaptic connections (i.e., neural plasticity) by degrading cell adhesion molecules and thus weakening neural extracellular scaffolds. Subsequently, this allows for the formation of new synaptic connections resulting in new neural pathways^20–22^. In AD, MMP-9 has been extensively studied due to its potential role in amyloid clearance, since it was shown to degrade Aβ peptides *in vitro* and *in vivo* ^23–25^. In this aspect we have previously reported that MMP-9 has a neuroprotective role *in vitro* and *in vivo*, by decreasing the formation of Aβ oligomers and by increasing the levels of neurotrophic factors, such as BDNF^26–29^.

In the present study we examined the role of MMP-9 on key components of the insulin survival pathway. Our experimental approaches included examination of these components in primary hippocampal cell cultures isolated from wild type, TgMMP-9, 5XFAD and 5XFAD/TgMMP-9 mice and in hippocampal extracts from 3 month-old animals of the same genotype. Our data demonstrate that overexpression of MMP-9 exerts beneficial effects by preventing the AD-related impairment of the insulin survival pathway in early stages of AD.

## Results

### Overexpression of MMP-9 enhanced IRS1 activation

**Figure 1:**
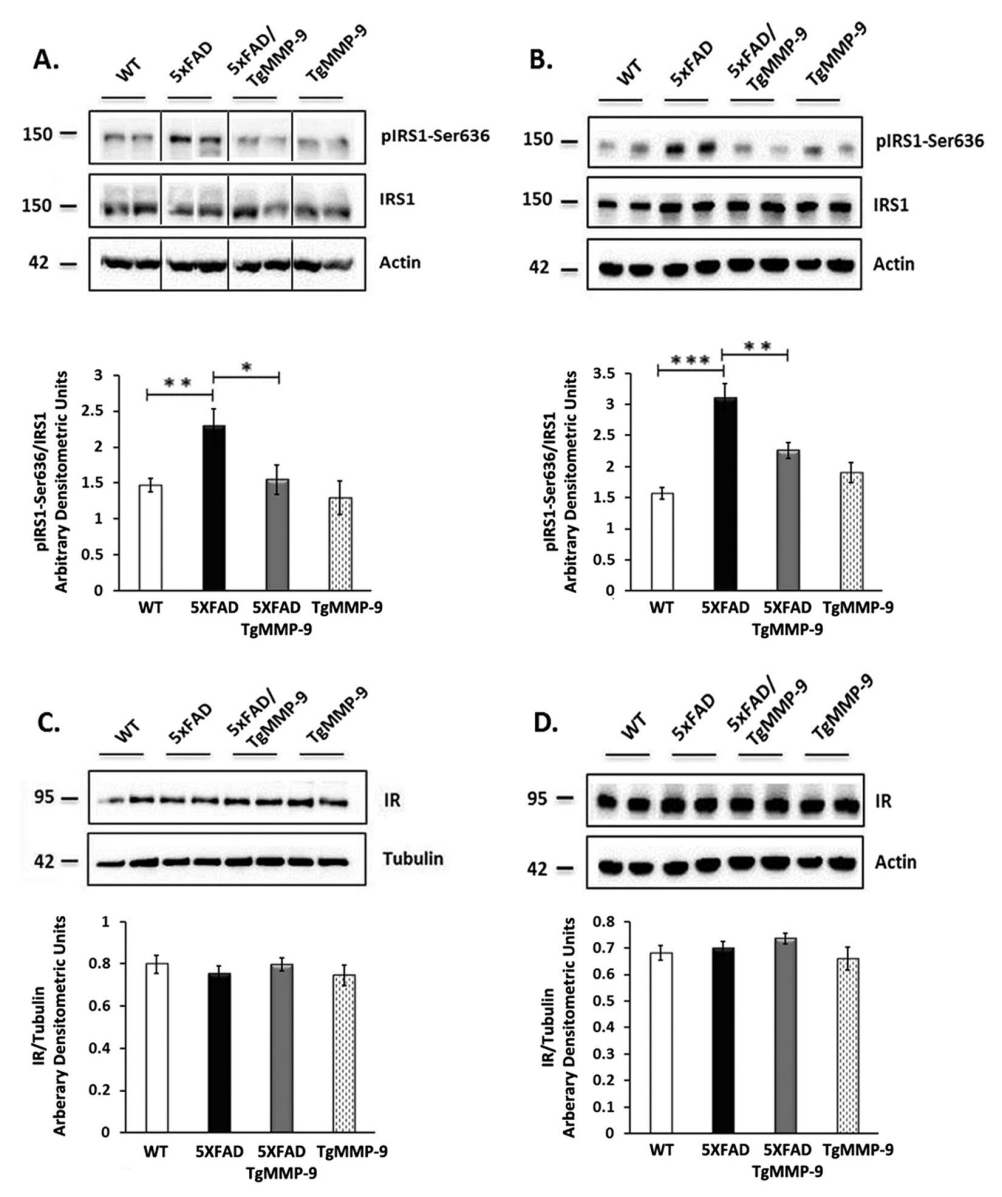
Representative Western blots of protein samples from primary hippocampal cell lysates (**A**,**C**) and hippocampal homogenates from 3 month-old mice (**B**,**D**) of WT, 5XFAD, 5XFAD/TgMMP-9 and TgMMP-9 mice. Equal amounts of total protein were analysed on 7.5% SDS-PAGE gels and immunoblotted with primary antibodies against pIRS1-Ser636, IRS1 and IR. To ensure equal loading, membranes were re-probed against β-Tubulin or β-Actin. Graphs depict densitometric quantification of phosphorylated proteins normalized to their respective total protein levels and total protein levels to their housekeeping gene. *n* = 8–12 (**A**), n = 11–14 (**C**), *n* = 7–10 (**B**), *n* = 5–12 (**D**). All data are presented as mean ± SEM (*p < 0.05, **p < 0.01, ***p < 0.001). Lanes were run on the same gel but were noncontiguous. Full-length images are presented in the Supplementary Information.

IRS1 phosphorylation is a critical step for the normal function of the insulin survival pathway. When IRS1 is phosphorylated at Tyrosine residues the insulin pathway is activated, whereas when it is phosphorylated at Serine residues its activation is inhibited, thus inhibiting the IR-mediated survival signaling. We initially investigated the phosphorylation levels of IRS1 at Serine-636 (pIRS1-Ser636) in all mouse genotypes. The results in Fig. 1 demonstrate that 5XFAD mice had significantly increased levels of pIRS1-Ser636 compared to their wild type littermates in both primary hippocampal cell cultures (Fig. 1A) and hippocampal extracts from 3 month-old mice (Fig. 1B). This effect, however, was not observed in MMP-9 overexpressing mice where pIRS1-Ser636 levels were reduced in 5XFAD/TgMMP-9 and TgMMP-9 mice to normal levels. No differences in the expression of total IRS1 was observed in primary neuronal cultures or in the hippocampal extracts of all types of mice examined. Similarly, no changes were detected in the expression of IR in all four genotypes (Fig. 1C,D).

**Figure 2:**
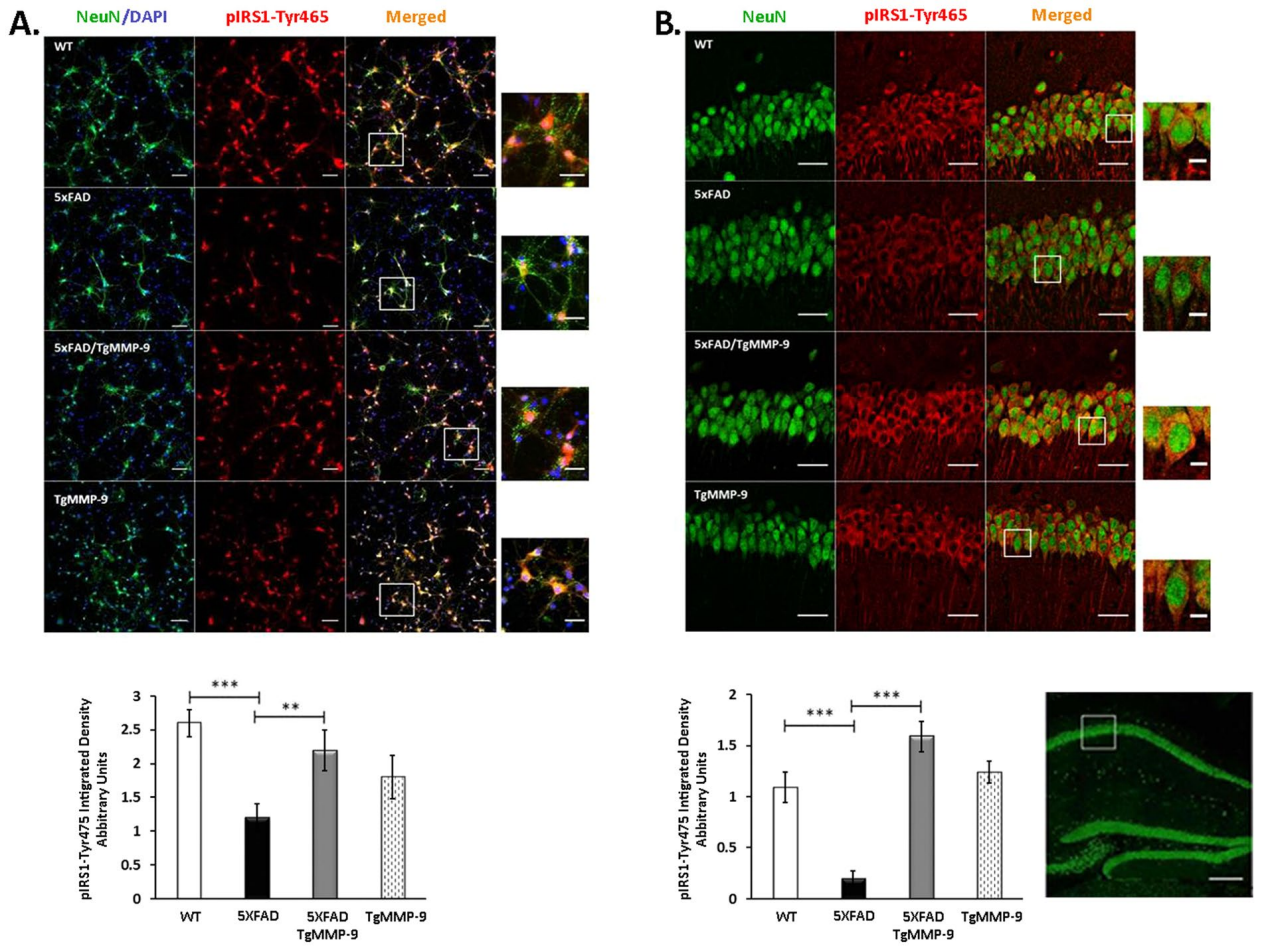
Representative images of primary hippocampal cultures (**A**) and 3 month-old mice CA1 hippocampal area (**B**) of WT, 5XFAD, 5XFAD/TgMMP-9 and TgMMP-9 mice labelled against pIRS1-Tyr465 antibody, depicted in red. Cell nuclei labelled with DAPI are depicted in blue and neurons labelled with NeuN are depicted in green. (**A**) Graph depicts quantification by the integrated density of total pIRS1-Tyr465. Magnification 20x, Scale bar: 50 μm. Scale bar of white box highlighted areas: 20 μm. (**B**). Graph depicts quantification by the integrated density of total pIRS1-Tyr465. Magnification 63x, Scale bar: 25 μm. Scale bar of white box highlighted areas: 5 μm. Hippocampal overview: Magnification 10x, Scale bar: 250 μm. *n* = 3 (**A**), *n* = 3 (**B**). All data are presented as mean ± SEM (**p < 0.01, ***p < 0.001).

In parallel, and consistent with the expected insulin resistance which was associated with pIRS1-Ser636, a significant decrease in activating phosphorylation levels of IRS1 at Tyrosine-465 (pIRS1-Tyr465) was observed. Immunofluorescence labeling of NeuN-positive hippocampal neurons isolated from 5XFAD mice revealed 50% reduction in the levels of pIRS1-Tyr465 compared to the wild type mice, whereas in the presence of MMP-9 in 5XFAD/TgMMP-9 and TgMMP-9 mice, pIRS1-Tyr465 phosphorylation remained within normal range (Fig. 2A). Similar results were obtained *in situ* by immunofluorescence labeling of the CA1 hippocampal area of 3 month-old mice which was also stained with anti-NeuN antibody, thus confirming the presence of pIRS1-Tyr465 in neuronal cells (Fig. 2B).

### MMP-9 overexpression increased Akt and GSK-3β phosphorylation levels

**Figure 3:**
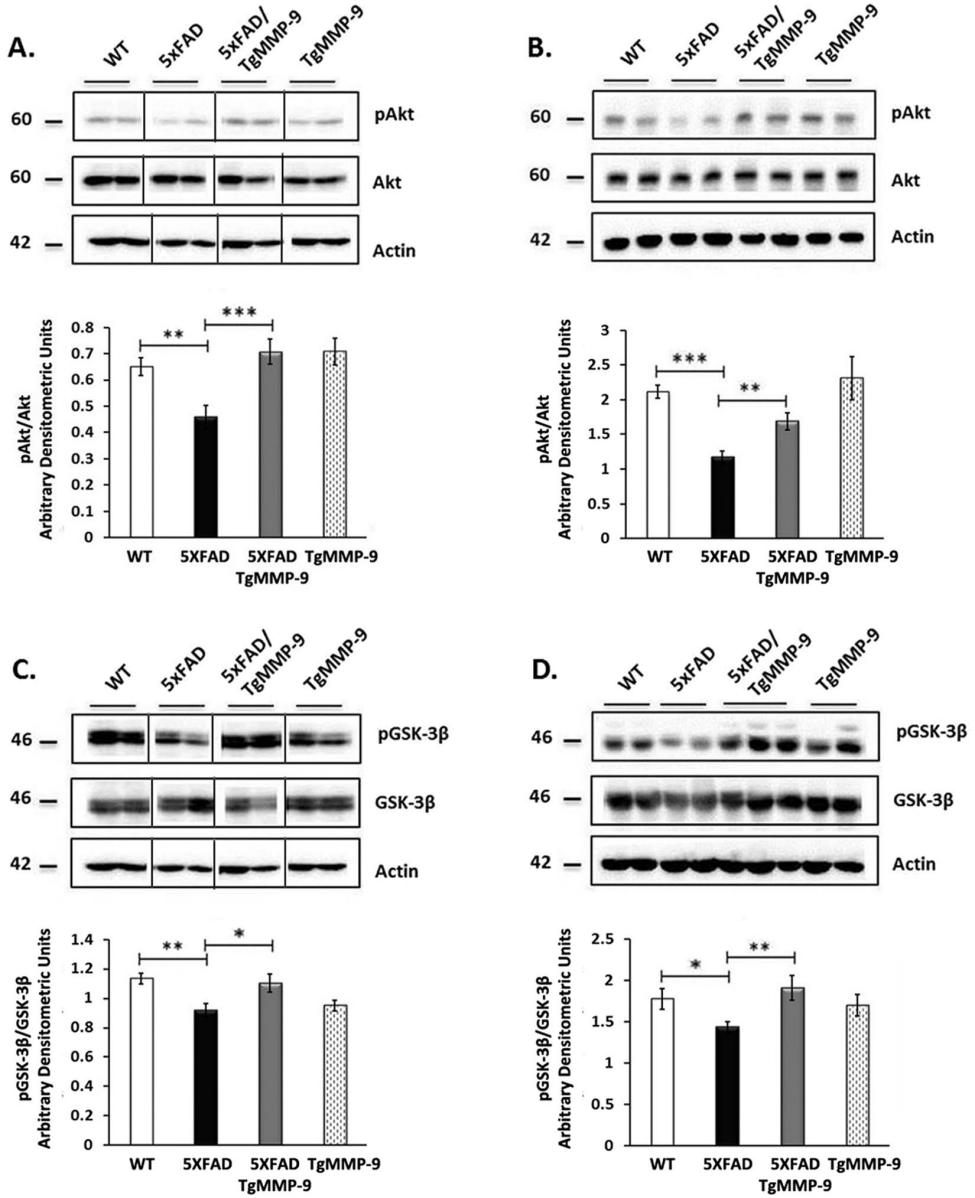
Representative Western blots of protein samples from primary hippocampal cell lysates (**A**,**C**) and hippocampal homogenates from 3 month-old mice (**B**,**D**) of WT, 5XFAD, 5XFAD/TgMMP-9 and TgMMP-9 mice. Equal amounts of total protein were analysed on 7.5% SDS-PAGE gels and immunoblotted with primary antibodies against pAkt and Akt (**A**,**B**), pGSK-3β and GSK-3β (**C**,**D**). To ensure equal loading, membranes were re-probed against β-Actin. Graphs depict densitometric quantification of phosphorylated proteins normalized to their respective total protein levels. *n* = 15–20 (**A**), n = 8–11 (**C**), *n* = 6–15 (**B**), *n* = 6–14 (**D**). All data are presented as mean ± SEM (*p < 0.05, **p < 0.01, ***p < 0.001). Lanes were run on the same gel but were noncontiguous. Full-length images are presented in the Supplementary Information.

Downstream the IR pathway, modulation of IRS1 affects directly the PI3-K/Akt signaling, which is pivotal for cell survival and normal cell function^30^. Decreased Akt activity was reported to reduce GSK-3β phosphorylation, which is implicated in neurodegeneration in AD^31^. To investigate the effect of MMP-9 in signaling kinases downstream the IRS1, we examined the phosphorylation levels of Akt at Serine 473 (pAkt) and GSK-3β-pSer9 (pGSK-3β) in primary hippocampal neurons (Fig. 3A,C) and in hippocampal extracts of 3 month-old mice (Fig. 3B,D). As shown in Fig. 3, a significant decline in hippocampal pAKT and pGSK-3β levels was observed in 5XFAD mice compared to wild type mice. In MMP-9 overexpressing mice however, this decrease in Akt and GSK-3β phoshorylation was prevented.

### MMP-9 overexpression is linked with TrkB activation via increased expression of BDNF

**Figure 4:**
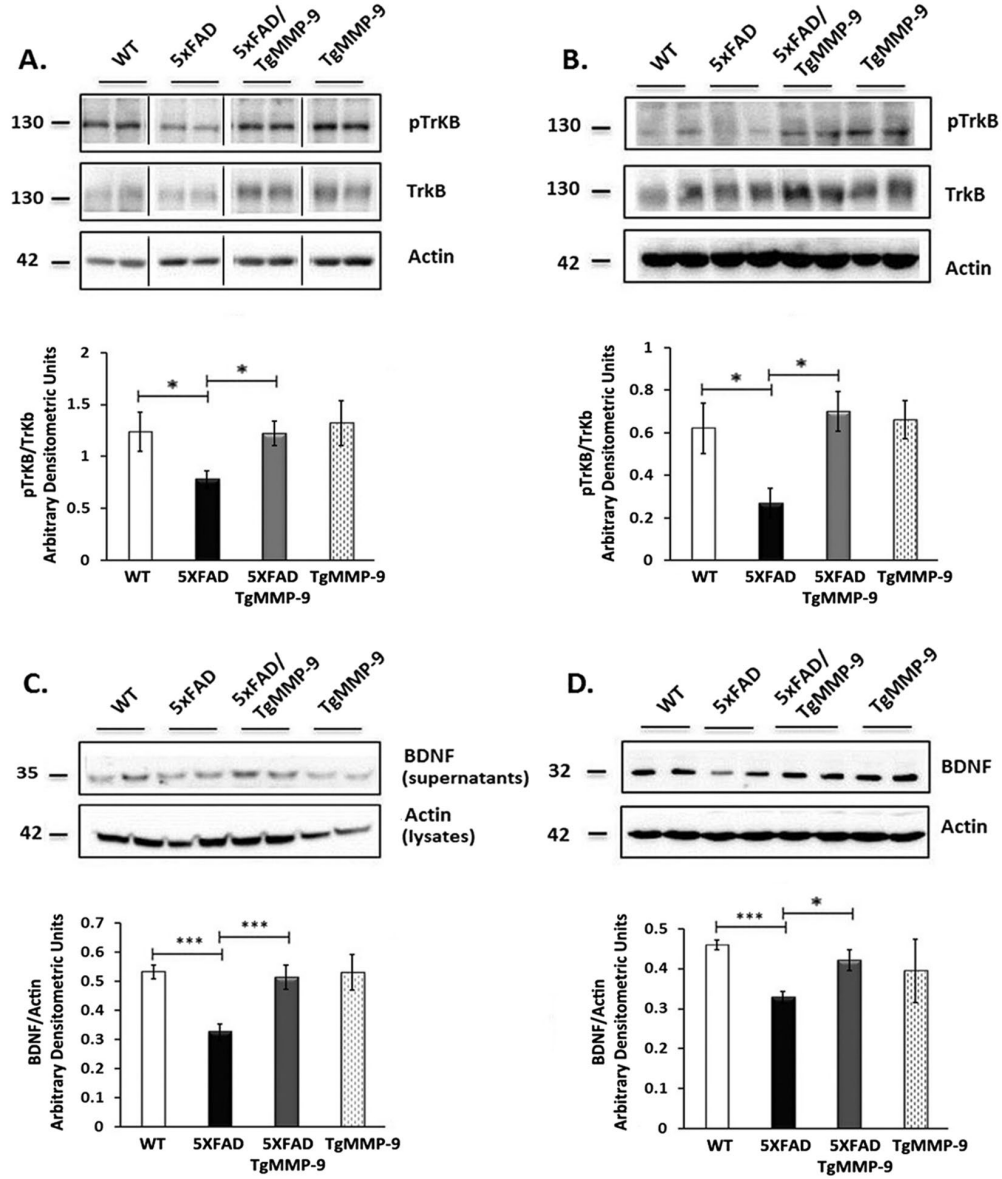
Representative Western blots of protein samples from primary hippocampal cultures (**A**,**C**) and hippocampal homogenates from 3 month-old mice (**B**,**D**) of WT, 5XFAD, 5XFAD/TgMMP-9 and TgMMP-9 mice. Equal amounts of total protein were analysed on 7.5% SDS-PAGE gels and immunoblotted with primary antibodies against pTrkB and TrkB (**A,B**). Equal amounts of total protein from hippocampal soluble fractions from 3 month-old mice and primary cell supernatants were analysed on 16% SDS-PAGE gels and immunoblotted against BDNF (**C**,**D**). Graphs depict densitometric quantification of phosphorylated proteins normalized to their respective total protein levels. To ensure equal loading, membranes were re-probed against β-Actin. *n* = 4–6 (**A**), *n* = 5–6 (**C**), *n* = 3 (**B**), *n* = 4 (**D**). BDNF levels from primary supernatants were corrected against actin from their respective cell lysates. All data are presented as mean ± SEM (*p < 0.05, ***p < 0.001). Lanes were run on the same gel but were noncontiguous. Full-length images are presented in the Supplementary Information.

It has previously been reported that MMP-9 overexpression leads to maturation of neurotrophic BDNF, an activator of the Tropomyosin B receptor (TrkB)^32^. Moreover, IRS1 activation has been shown to be also regulated by Trk activation^33^. Hence, we examined whether activation of TrkB by Tyrosine phosphorylation due to overexpression of MMP-9 mediates the activation of IRS1. The obtained data indicated that in 5XFAD mice, phosphorylation of TrkB was significantly reduced compared to wild type mice, while its phosphorylation levels were maintained to normal levels in samples from TgMMP-9 and 5XFAD/TgMMP-9 both in primary hippocampal neurons (Fig. 4A) and in hippocampal extracts from 3 month-old animals (Fig. 4B). In sequence, we examined changes in the levels of BDNF. The data presented in Fig. 4 demonstrate that in primary hippocampal cells (Fig. 4C) and hippocampal extracts (Fig. 4D) of 5XFAD animals, significantly lower levels of BDNF were detected compared to wild type animals, an effect that was aborted in 5XFAD/TgMMP-9 mice.

**Figure 5:**
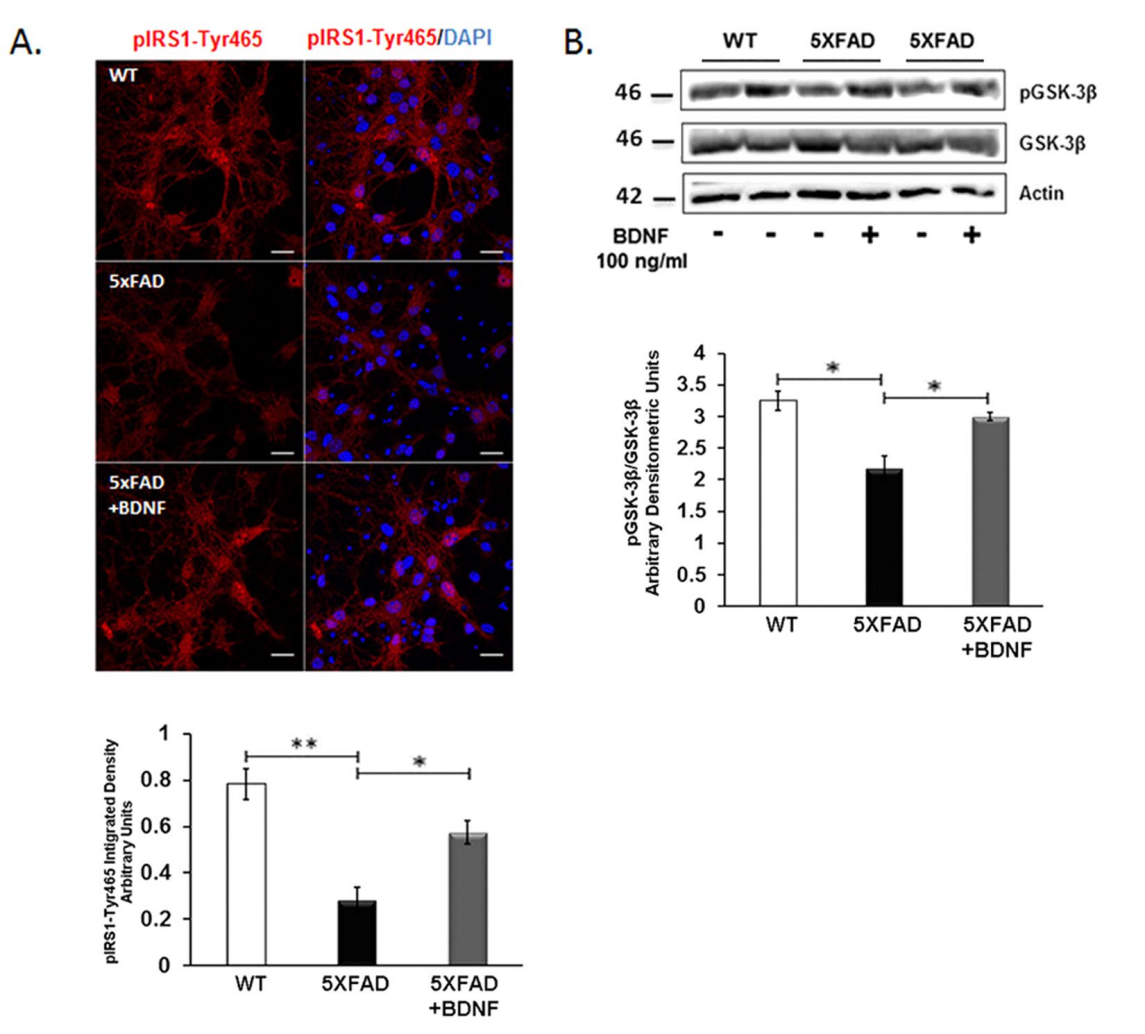
Primary hippocampal cultures of 5XFAD mice were treated with 100 ng/ml of recombinant BDNF for 30 min. (**A**) Cells labelled against pIRS1-Tyr465 antibody are depicted in red and cell nuclei labelled with DAPI are depicted in blue. Graph depicts quantification by the integrated density of total pIRS1-Tyr465. Magnification 63x, Scale bar: 20 μm. (**B**) Equal amounts of total protein from treated cells were analyzed on 10% SDS-PAGE gels and immunoblotted with primary antibodies against pGSK-3β and GSK-3β. To ensure equal loading, membranes were re-probed against β-Actin. Graphs depict densitometric quantification of phosphorylated proteins normalized to their respective total protein levels. *n* = 3 (**A**) and n = 4 (**B**). All data are presented as mean ± SEM (*p < 0.05, **p < 0.01).

To further prove that the activation of IRS1 is modulated by BDNF directly, 100 ng/ml of recombinant BDNF was added to primary cultured cells of 5XFAD mice and pIRS1-Tyr465 and pGSK3-β levels were compared to untreated cells of animals of the same genotype. We observed a significant increase in pIRS1-Tyr465 levels (Fig. 5A) followed by an increase in the levels of pGSK3-β (Fig. 5B) compared to untreated cells.

### MMP-9 overexpression reduced hippocampal phosphorylation of JNK and protected against cell apoptosis

**Figure 6:**
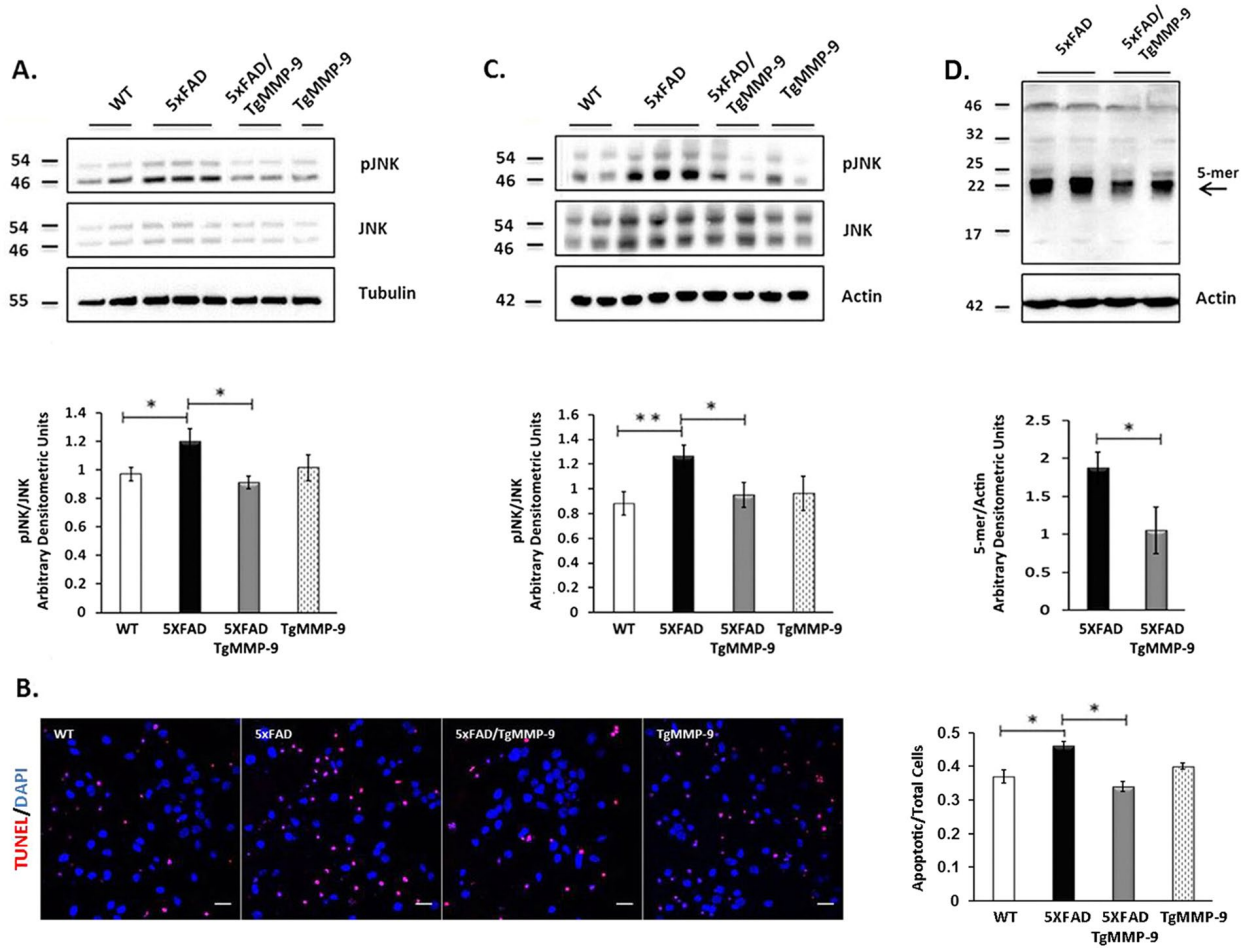
Representative Western blots of protein samples from primary hippocampal cell lysates (**A**) and hippocampal homogenates from 3 month-old mice (**C**) of WT, 5XFAD, 5XFAD/TgMMP-9 and TgMMP-9 mice. Equal amounts of total protein were analysed on 7.5% SDS-PAGE gels and immunoblotted with primary antibodies against pJNK and JNK. To ensure equal loading, membranes were re-probed against β-Actin or β-Tubulin. Graphs depict densitometric quantification of phosphorylated proteins normalized to their respective total protein levels. (**B**) Representative images of primary hippocampal cultures of WT, 5XFAD, 5XFAD/TgMMP-9 and TgMMP-9 mice labelled with TUNEL staining. Apoptotic cells are depicted in red and cell nuclei with stained with DAPI are depicted in blue. Graph depicts apoptotic cell number relative to total cell number. Magnification 63x. Scale bar: 20 μm. (**D**) Equal amounts of total protein from hippocampal soluble fractions from 3 month-old 5XFAD and 5XFAD/TgMMP-9 mice were analysed on 16% SDS-PAGE gels and immunoblotted with primary antibody against human anti-6E10. *n* = 6–7 (**A**), *n* = 3 (**B**), *n* = 6–10 (**C**), *n* = 3 (**D**). All data are presented as mean ± SEM (*p < 0.05, **p < 0.01). Lanes were run on the same gel but were noncontiguous. Full-length images are presented in the Supplementary Information.

In peripheral insulin resistance, JNK is one of the most important stress-activated kinases, which is involved in IRS1 Serine phosphorylation^34^. Similarly to these mechanisms, a key role of JNK activation in neuronal insulin resistance in the presence of Aβ oligomers was recently demonstrated^35^. We previously reported that overexpression of MMP-9 significantly reduced Aβ oligomers in the brain of 7 month-old 5XFAD mice^29^. Thereby, we investigated the effect of MMP-9 overexpression on the phosphorylation of JNK in our examination system. As shown in Fig. 6, hippocampal levels of pJNK were significantly increased in cultured primary neurons of 5XFAD mice (Fig. 6A), an effect accompanied with increased cell apoptosis compared to wild type mice, as detected with TUNEL assay (Fig. 6B). In cultured cells of 5XFAD/TgMMP-9 mice, however, JNK phosphorylation levels remained within normal levels and cell apoptosis was significantly lower compared to 5XFAD mice (Fig. 6A,B). Similarly, in hippocampal extracts from 3 month-old mice of 5XFAD/TgMMP-9 mice, we detected significantly lower pJNK levels compared to 5XFAD mice (Fig. 6C), presumably attributed to a significant reduction in Aβ oligomers levels (Fig. 6D).

## Discussion

Insulin survival pathway impairment caused by Aβ oligomers is considered a key event in the pathogenesis of AD during the early stages of the disease^17^. Therefore, there is a growing need to develop strategies in order to counterbalance their compromising effects on insulin survival-related proteins. In the present study, we suggest a dual mechanism of neuroprotective action which is attributed to overexpression of MMP-9.

The obtained data indicate that in 5XFAD mice, which are well studied animal models of AD, the IR-mediated insulin survival pathway is severely compromised due to halting of IRS1 activity. This effect is caused by an increase in phosphorylation at pIRS1-Ser636 and a respective decrease in phosphorylation in pIRS1-Tyr465, without affecting IRS1 levels. Our observations are in accordance with findings from a previous study, in which treatment of primary rat hippocampal cell cultures with exogenous Aβ oligomers lead to enhanced pIRS1-Ser636 with a concomitant reduction of pIRS1-Tyr465^35^. pIRS1-Ser636 was also observed to be substantially enhanced in our samples from 5XFAD mice. This effect was prevented in the 5XFAD/TgMMP-9 mouse model, indicating that MMP-9 either provides a stimulus towards IRS1 activation via pIRS1-Tyr465 or impedes the inhibitory phosphorylation of IRS1 by interfering with the formation of Aβ oligomers.

It is worth noting that phosphorylation levels of IRS1 at Ser636 or Tyr465 were not changed in the TgMMP-9 mouse compared to its wild type littermate, indicating that enhanced expression of MMP-9 did not suffice by itself to cause IRS1 modifications. Moreover, the expression of IRS1 or IR was not significantly altered in TgMMP-9 or 5XFAD/TgMMP-9 mice. This finding further indicates that MMP-9 does not exert its action via directly interacting with IR or IRS1. Furthermore, previous studies showed that in primary hippocampal cultures total IR levels were not changed following treatment with Aβ oligomers; the suggestion was made that a redistribution of these toxic oligomers could facilitate the observed increase of pIRS1-Ser636^13, 14^. The data obtained from our experiments from the group of 3 month-old animals agree with previous studies, in which the levels of total IR remain unchanged until the age of 12 months in AD mouse models, thus indicating that the expression of IR is suppressed in later stages of the disease^36^. Additionally, observations from other studies suggested that reduced expression of IRS1 occurred after the age of 9 months in AD mice^37^.

Further evidence of impairment downstream of the pathway was demonstrated by inhibition of pAkt which lead to the activation of GSK-3β kinase, as suggested by the observed significant reduction of GSK-3β phosphorylation. The obtained data reinforce previous studies, according to which, neurons treated with exogenous Aβ exhibited similar effects^38^. Moreover, our experiments documented that decreased GSK-3β phosphorylation which accompanies AD, was prevented both *in vitro* and *in vivo* in 5XFAD/TgMMP-9 mice, since unperturbed levels of pAkt and pGSK-3β kinases were observed. It appears then, that the presence of enhanced amounts of MMP-9 during development and early in life preserves normal function of the insulin survival pathway in our experimental animal model.

In order to address the significant increase of pIRS1-Tyr465 in 5XFAD/TgMMP-9 mice, we investigated whether MMP-9 can activate IRS1 by Tyrosine phosphorylation. Since MMP-9 did not alter the expression levels of IR or IRS1, we investigated modulation of IRS1 by other factors. Previous reports have demonstrated that in primary neurons IRS1 phosphorylation was linked to the PI3K/Akt pathway through the action of BDNF^39, 40^. Moreover, in previous studies by us and others it was demonstrated that MMP-9 mediated the conversion of pro-BDNF to mature BDNF^20, 29, 41^ and that reduced BDNF expression was observed in 5XFAD mice at the age of 7 months^42^. In the experiments described herein, we observed decreased levels of BDNF in 3 month-old 5XFAD mice compared to the wild type. However, in 5XFAD/TgMMP-9 mice, BDNF was increased in both primary cultures and hippocampal extracts from 3 month-old animals. Increased BDNF levels lead to activation of BDNF receptor, TrkB, by phosphorylation. pTrkB was significantly enhanced in 5XFAD/TgMMP-9 compared to 5XFAD samples, thus leading to IRS1 activation. To further strengthen our hypothesis that MMP-9 exerts its action via BDNF increase, we treated neurons from 5XFAD mice with exogenous BDNF and observed enhanced activation of the pathway upstream, indicated by increased pIRS1-Tyr465 levels, as well as downstream, indicated by increased pGSK-3β levels, compared to untreated cells.

Furthermore, we examined whether MMP-9 overexpression directly interferes with the formation of Aβ oligomers which have been documented to lead to JNK activation. It was previously reported that accumulation of Αβ oligomers in the synaptic area resulted in JNK activation, which in turn lead to pIRS1-Ser636 phosphorylation and to insulin resistance^43^. Moreover, it was demonstrated that treatment of primary neuronal cultures with Aβ oligomers resulted in dose-dependent cell toxicity as a result of JNK phosphorylation^44^. Additionally, increased JNK phosphorylation was also observed in post-mortem brains of AD patients^45, 46^, as well as in an AD mouse model^47^. Our experiments also indicated significantly increased pJNK in 5XFAD mice, which *in vitro*, resulted in increased apoptosis compared to wild type mice. The observed increase in pJNK levels was accompanied, by significantly increased pIRS1-Ser636, both *in vitro* and *in vivo*. In contrast, pJNK levels were not altered in 5XFAD/TgMMP-9 and TgMMP-9 mice compared to the wild type, which is in accordance with low pIRS1-Ser636 levels observed in these mice. This finding suggests that MMP-9 inhibited pIRS1-Ser636, in part by interfering with the formation of Aβ oligomers. Our previous results support the suggestion of an inhibitory effect of MMP-9 on Aβ formation, since we demonstrated that in aged, 7 month-old 5XFAD/TgMMP-9 mice, Aβ oligomers were significantly decreased compared to 5XFAD mice of the same age^29^. In this report, we examined the amount of Aβ oligomers in hippocampal soluble extracts from young, 3 month-old 5XFAD and 5XFAD/TgMMP-9 mice. Reduced levels of the 5-mer (20 kDa) amyloid peptide in 5XFAD/TgMMP-9 samples were observed compared to 5XFAD. Apparently then, MMP-9 interferes with the generation of Aβ oligomers during early stages of AD in our experimental model. This finding suggests that Aβ oligomers compromise insulin survival and adversely affect neuronal cell function as early as at the age of three months, before the manifestation of the AD symptoms.

**Figure 7:**
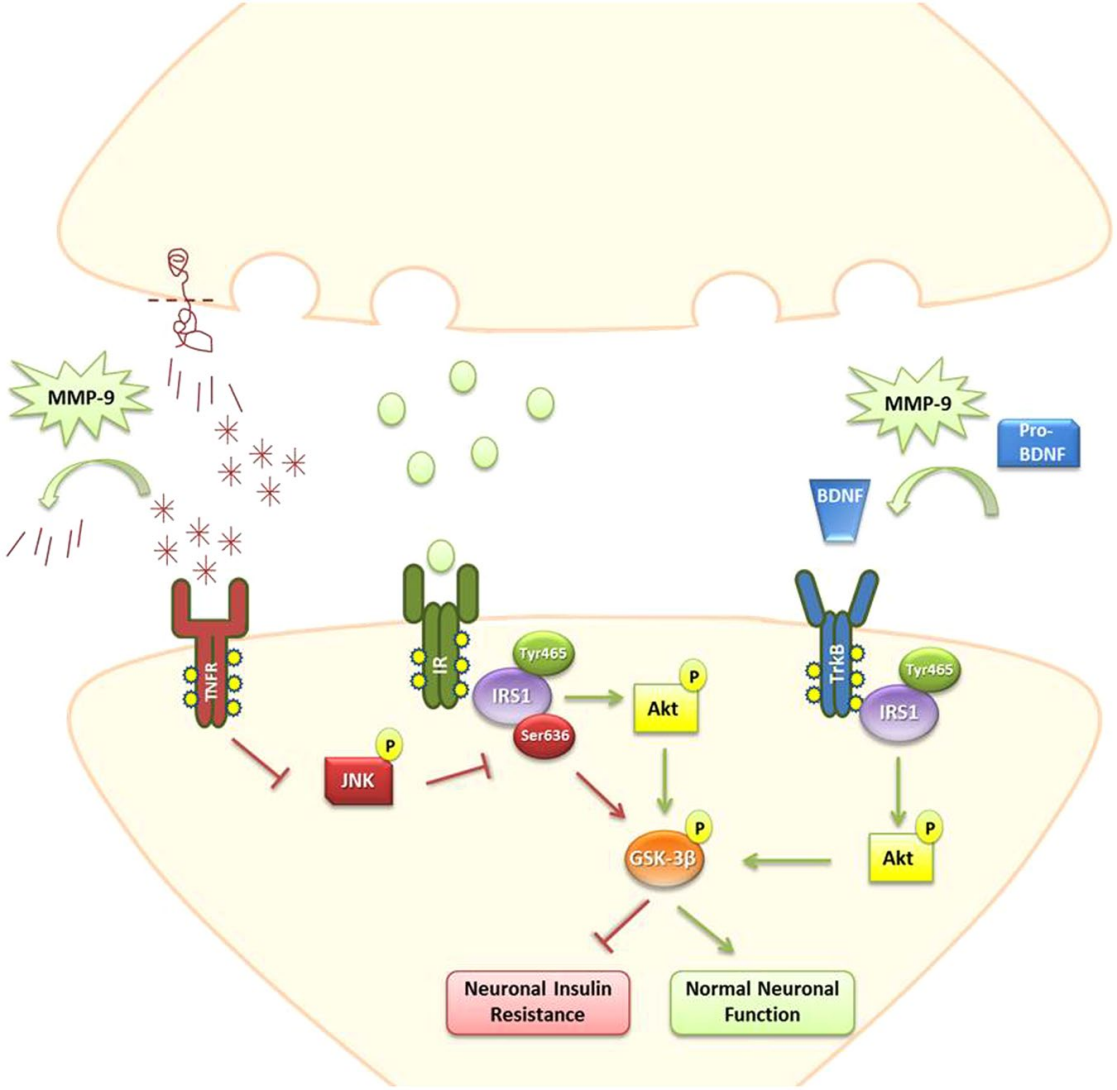
Proposed mechanism of neuroprotective action of MMP-9 in preventing Aβ-mediated impairment of the insulin survival pathway. Aβ oligomers generated during early stages of AD stimulate pro-apoptotic pathways, such as TNFR signaling, which activates JNK kinase, resulting in neuronal insulin resistance via IRS1-Ser636 phosphorylation and GSK-3β activation. MMP-9 protects, in part, by reducing Aβ amyloid peptide accumulation, thus blocking Aβ oligomeric-induced defects in insulin signaling. MMP-9 also provides an additional stimulus to insulin, by increasing BDNF levels. The latter binds to the TrkB receptor, inducing IRS1-Tyr465 phosphorylation and Akt activation, hence contributing to normal neuronal function.

Overall, our results suggest that overexpression of MMP-9 prevents, at least in part, the impairment of the insulin survival pathway *in vitro* in primary hippocampal cell cultures of 5XFAD animals, as well as *in vivo* in young animals of the same genotype. To our knowledge, it is the first time that impairment of insulin survival pathway is been reported to occur early during development of AD in an experimental animal model. A proposed mechanism suggested by our data depicts a dual way of action of MMP-9: MMP-9 acts a) by reducing Aβ oligomers that activate the pro-apoptotic JNK which triggers IRS1-Ser636 phosphorylation and b) by facilitating IRS1-Tyr465 phosphorylation via increasing BDNF levels (Fig. 7). This mechanism is further supported by the documented decrease of pJNK and increase of pAkt, pGSK-3β and pTrkB, both *in vitro* and *in vivo*, in 5XFAD/TgMMP-9 samples, compared to their 5XFAD counterparts. We conclude that the presence of enhanced amounts of MMP-9 facilitates the unperturbed function of insulin-mediated survival, which is compromised during early stages of AD.

## Methods

### Experimental animals

5XFAD and TgMMP-9 mice on a C57BL/6 background were used in all experiments. The generation of 5XFAD and TgMMP-9, respectively, has been previously reported^28, 48^. Briefly, 5XFAD mice co-express and co-inherit FAD mutant forms of human APP [Swedish (K670N, M671L), Florida (I716V) and London (V717I) mutation] and presenilin-1 (M146L; L286V) transgenes under the transcriptional control of the neuron-specific mouse Thy-1 promoter, whereas TgMMP-9 mice express the wild-type human pro-MMP-9 sequence under the transcriptional control of the neuron-specific promoter platelet-derived growth factor (PDGF)-β. 5XFAD and TgMMP-9 mice were both kept in a heterozygote state and for the generation of 5XFAD/TgMMP-9 mice, TgMMP-9 female mice were crossed with 5XFAD male mice^29^. Age-matched wild type mice on the same background were used as controls.

Genotyping was performed by PCR analysis of tail DNA using primers specific for human MMP-9 gene (forward: 5′-GCACCACCACAACATCACCTAT-3′, reverse: 5′-AAACTGGATGACGATGTCTGCG-3′) and the human APP gene (forward 5′-GAATTCCGACATGACTCA-3′, reverse: 5′-GTTCTGCTGCATCTTGGACA-3′). PCR reactions were performed for 30 cycles at 95 °C for 1 min, 60 °C for 1 min and 72 °C for 1 min. All necessary reagents were purchased from Invitrogen (Carlsbad, CA, USA) and Nippon (Dueren, Germany). All animals were kept under standard conditions (24 °C, 12-h light/dark cycle, lights on at 8:00 AM), housed in mixed genotype groups and received food and water ad libitum. All animal procedures were performed in compliance with European legislation while they were approved by the Bioethical committee of the Bioscience and Application Institute of NCSR “Demokritos” and by the Hellenic Ministry of Rural Development and Food (Animal welfare assurance number: GR equivalent: EL25 BIO 019, 020, 022).

### Primary hippocampal cultures and treatment with BDNF

Primary hippocampal cultures were established based on previously described protocols^49, 50^. Briefly, p0-3 postnatal mice were euthanized and their hippocampi were isolated. The tissue was enzymatically digested with Trypsin/EDTA 0.25%, 0.02% (Biochrom, Berlin, Germany) for 20 min at 37 °C. Trypsin was deactivated with Hibernate-A medium containing 10% fetal bovine serum (FBS) and the tissue was carefully rinsed several times with plain Hibernate-A to remove any traces of serum. After proper trituration, the cells were seeded on poly-D-lysine (Sigma-Aldrich, St. Louis, MO, USA) 24-well plates at a density of 350.000 cells per well, in a manner of two wells per animal of a different genotype. The cells were cultured at 37 °C, in humidified 5% CO_2_, 95% atmosphere in Neurobasal-A medium supplemented with 2% B-27, 0.5 mM Glutamax and 1% penicillin/streptomycin, until proper differentiation. The medium was half-replaced every 3 days. For immunofluorescence experiments, cells were plated on poly-D-lysine coverslips on a density of 50.000 cells per well. All primary culture media and supplements were purchased from ThermoFisher Scientific Inc. (Rockford, IL, USA). For the BDNF treatment, primary hippocampal cells isolated from wild type and 5xFAD mice were cultured until differentiation and were then treated with 100 ng/ml recombinant BDNF (R & D systems, Minneapolis, MN, USA) in complete Neurobasal-A medium for 30 minutes, prior to cell lysis and fixation.

### Protein extraction

Primary hippocampal cells were lysed in RIPA buffer (Tris-HCl 50 mM, NaCl 150 mM, EDTA 1 mM, Na deoxycolate 0.25%, TritonX-100 1%, SDS 0.1%) containing a protease and phosphatase inhibitors cocktail (Roche, Mannheim, Germany). The lysates were incubated on ice for 1 hour and centrifuged for 15 minutes at 13.000 rpm at 4 °C. The supernatants were collected and the protein content was determined by the Bradford protein assay (ThermoFisher). 3 month-old animals of all four genotypes were euthanized and dissected hippocampi were homogenized with a mechanical pestle in RIPA buffer containing a protease and phosphatase inhibitor cocktail.

For the detection of soluble proteins, dissected hippocampi were initially homogenized in 5 volumes of ice-cold buffer (0.02 M Tris-HCl, pH 8.5) containing the same inhibitor cocktail and centrifuged for 1 hour at 47.000 rpm at 4 °C.

### Immunoblotting

Equal amounts of protein from cell lysates and hippocampal homogenates were separated on 7.5 or 16% SDS-PAGE gels and transferred onto nitrocellulose membranes. Membranes were blocked with 5% non-fat milk, 0.1% Tween-20 in TBS for 1 hour at room temperature and incubated overnight at 4 °C with primary antibodies, anti-pIRS1-Ser636, anti-IRS1, anti-IR, anti-BDNF in dilution 1:500 (Santa Cruz Biotechnology, Santa Cruz, CA, USA), anti-pAkt, anti-Akt, anti-pGSK-3β, anti-GSK-3β, anti-pJNK, anti-JNK in dilution 1:1000 (Cell signalling Technology, Danvers, MA, USA) and anti-6E10 in dilution 1:1000 (BioLegend, San Diego, CA, USA), according to manufacturers’ instructions. Membranes were washed in TBS/Tween-20 and incubated with horseradish peroxidase-conjugated secondary anti-rabbit and anti-mouse antibodies in dilution 1:1000 (GE healthcare, Buckinghamshire, UK) for 1 hour at room temperature. Blots were visualized with an ECL chemiluminescence substrate reagent kit (ThermoFisher).

Quantification of protein levels was performed by densitometry of the immunoreactive bands by ImageJ software (National Institutes of Health, Bethesda, MD, USA). Total protein levels of IR, IRS1, Akt, JNK, GSK-3β, 6E10 and BDNF were corrected by housekeeping β-Tubulin or β-Actin (Sigma-Aldrich) after re-probing. Quantification of phosphorylated protein levels was determined after normalization to their respective total protein levels.

### Immunofluorescence

Primary hippocampal cells on coverslips were fixed with 4% Paraformaldehyde (PFA)/Phosphate Buffer Saline (PBS) for 20 min at room temperature, rinsed with PBS, permeabilized with 0.3% Triton X-100 for 10 min and blocked with 10% FBS in PBS for 2 hours at room temperature. Following that, cells were incubated overnight at 4 °C with primary antibody anti-pIRS1-Tyr465 (Santa Cruz) in dilution 1:200 and anti-NeuN (Merck Millipore, Temecula, CA, USA) in dilution 1:500 in 5% FBS in PBS and with secondary antibodies, anti-rabbit AlexaFluor594 and anti-mouse AlexaFluor488 (ThermoFisher) in 5% FBS in PBS for 1 hour at room temperature. For nuclei visualization cells were incubated with DAPI for 5 min. Finally, cells on coverslips were mounted with mounting medium (Dako, Denmark) and were observed with a Leica DFC-500 fluorescence microscope. For immunofluorescence 10–15 images were collected from 3 independent batches of culture of all four different genotypes. For the BDNF peptide treatment, cells were observed with a Leica TCS SP8 MP, inverted confocal microscope.

The acquired images were quantified by measuring the integrated density of each image corrected by the number of cells per frame with ImageJ software. Appropriate threshold was applied to all conditions.

### Immunohistochemistry

3 month-old mice of all four genotypes were euthanized and their hemi-brains were isolated, rinsed in cold PBS and fixed in 4% PFA/PBS. Serial 50 μm thick brain sections were obtained, using Vibratome (Leica, VT1200S). Immunohistochemistry was performed using free-floating sections in cold PBS. Sections were blocked in 10% FBS, 0.3% Triton-X100/PBS for 2 hours at room temperature. The slices were incubated with anti-pIRS1-Tyr465 in dilution 1:100 and anti-NeuN in dilution 1:500, followed by incubation with anti-rabbit AlexaFluor594 and anti-mouse AlexaFluor488 in 5% FBS, 0.1% Triton-X100/PBS, as described in the immunocytochemistry section. Finally, sections were mounted in Dako mounting medium and were imaged with a Bio-Rad (MRC 1024 ES) confocal microscope. The acquired images from the CA1 hippocampal area were quantified by measuring the integrated density of each image corrected by the number of cells per frame with ImageJ software. Appropriate threshold was applied to all conditions.

### TUNEL Assay

Apoptotic hippocampal neurons were detected using the *In Situ* Cell Death Detection Kit, TMR red (Roche) according to the manufacturer’s protocol. Briefly, primary hippocampal neurons were fixed with 4% Paraformaldehyde (PFA)/Phosphate Buffer Saline (PBS) for 20 min at room temperature, rinsed with PBS and permeabilized with 3% Triton X-100 for 10 min. Next, cells were incubated with TUNEL reaction mixture for 1 hour at 37 °C. Cell nuclei were visualized with DAPI stain. Finally, cells on coverslips were mounted with Dako mounting medium and were observed with a Leica TCS SP8 MP, inverted confocal microscope. 6–8 images were collected from 3 independent batches of culture of all four different genotypes. Apoptosis was calculated with ImageJ software by measuring the number of labeled red cells relative to the total cell number.

### Statistical Analysis

Data are presented as mean ± standard error of the mean (SEM). Three to four independent experiments were performed, with at least *n* = 3 per mouse genotype, specified for each set of experiments in the figure legends. For western blot experiments data were pooled from multiple membranes. Significance was defined as p < 0.05 by using a two-way ANOVA with Origin software (Origin Lab corporation, Northampton, MA, USA).

## Electronic supplementary material

Supplementary Information

## References

[1] Anand R, Gill KD, Mahdi AA (2014). Therapeutics of Alzheimer’s disease: Past, present and future. Neuropharmacology.

[2] Hardy J (2009). The amyloid hypothesis for Alzheimer’s disease: a critical reappraisal. Journal of neurochemistry.

[3] Cheng IH (2007). Accelerating amyloid-beta fibrillization reduces oligomer levels and functional deficits in Alzheimer disease mouse models. The Journal of biological chemistry.

[4] Mucke L (2000). High-level neuronal expression of abeta 1-42 in wild-type human amyloid protein precursor transgenic mice: synaptotoxicity without plaque formation. The Journal of neuroscience: the official journal of the Society for Neuroscience.

[5] Gong Y (2003). Alzheimer’s disease-affected brain: presence of oligomeric A beta ligands (ADDLs) suggests a molecular basis for reversible memory loss. Proceedings of the National Academy of Sciences of the United States of America.

[6] LaFerla FM, Green KN, Oddo S (2007). Intracellular amyloid-beta in Alzheimer’s disease. Nature reviews. Neuroscience.

[7] Ring S (2007). The secreted beta-amyloid precursor protein ectodomain APPs alpha is sufficient to rescue the anatomical, behavioral, and electrophysiological abnormalities of APP-deficient mice. The Journal of neuroscience: the official journal of the Society for Neuroscience.

[8] Chasseigneaux S, Allinquant B (2012). Functions of Abeta, sAPPalpha and sAPPbeta: similarities and differences. Journal of neurochemistry.

[9] Duarte AI, Moreira PI, Oliveira CR (2012). Insulin in central nervous system: more than just a peripheral hormone. Journal of aging research.

[10] Chiu SL, Chen CM, Cline HT (2008). Insulin receptor signaling regulates synapse number, dendritic plasticity, and circuit function *in vivo*. Neuron.

[11] de la Monte SM (2009). Insulin resistance and Alzheimer’s disease. BMB reports.

[12] Lacor PN (2007). Abeta oligomer-induced aberrations in synapse composition, shape, and density provide a molecular basis for loss of connectivity in Alzheimer’s disease. The Journal of neuroscience: the official journal of the Society for Neuroscience.

[13] Zhao WQ (2008). Amyloid beta oligomers induce impairment of neuronal insulin receptors. FASEB journal: official publication of the Federation of American Societies for Experimental Biology.

[14] De Felice FG (2009). Protection of synapses against Alzheimer’s-linked toxins: insulin signaling prevents the pathogenic binding of Abeta oligomers. Proceedings of the National Academy of Sciences of the United States of America.

[15] Hooper C, Killick R, Lovestone S (2008). The GSK3 hypothesis of Alzheimer’s disease. Journal of neurochemistry.

[16] Mines MA, Beurel E, Jope RS (2011). Regulation of cell survival mechanisms in Alzheimer’s disease by glycogen synthase kinase-3. International journal of Alzheimer’s disease.

[17] De Felice FG (2013). Alzheimer’s disease and insulin resistance: translating basic science into clinical applications. The Journal of clinical investigation.

[18] Talbot K (2012). Demonstrated brain insulin resistance in Alzheimer’s disease patients is associated with IGF-1 resistance, IRS-1 dysregulation, and cognitive decline. The Journal of clinical investigation.

[19] Egeblad M, Werb Z (2002). New functions for the matrix metalloproteinases in cancer progression. Nature reviews. Cancer.

[20] Ethell IM, Ethell DW (2007). Matrix metalloproteinases in brain development and remodeling: synaptic functions and targets. Journal of neuroscience research.

[21] Agrawal SM, Lau L, Yong VW (2008). MMPs in the central nervous system: where the good guys go bad. Seminars in cell & developmental biology.

[22] Michaluk P, Kaczmarek L (2007). Matrix metalloproteinase-9 in glutamate-dependent adult brain function and dysfunction. Cell death and differentiation.

[23] Backstrom JR, Lim GP, Cullen MJ, Tokes ZA (1996). Matrix metalloproteinase-9 (MMP-9) is synthesized in neurons of the human hippocampus and is capable of degrading the amyloid-beta peptide (1–40). The Journal of neuroscience: the official journal of the Society for Neuroscience.

[24] Yan P (2006). Matrix metalloproteinase-9 degrades amyloid-beta fibrils *in vitro* and compact plaques *in situ*. The Journal of biological chemistry.

[25] Hernandez-Guillamon M (2015). Sequential Amyloid-beta Degradation by the Matrix Metalloproteases MMP-2 and MMP-9. The Journal of biological chemistry.

[26] Talamagas AA, Efthimiopoulos S, Tsilibary EC, Figueiredo-Pereira ME, Tzinia AK (2007). Abeta(1–40)-induced secretion of matrix metalloproteinase-9 results in sAPPalpha release by association with cell surface APP. Neurobiology of disease.

[27] Fragkouli A, Tzinia AK, Charalampopoulos I, Gravanis A, Tsilibary EC (2011). Matrix metalloproteinase-9 participates in NGF-induced alpha-secretase cleavage of amyloid-beta protein precursor in PC12 cells. Journal of Alzheimer’s disease: JAD.

[28] Fragkouli A (2012). Enhanced neuronal plasticity and elevated endogenous sAPPalpha levels in mice over-expressing MMP9. Journal of neurochemistry.

[29] Fragkouli A, Tsilibary EC, Tzinia AK (2014). Neuroprotective role of MMP-9 overexpression in the brain of Alzheimer’s 5XFAD mice. Neurobiology of disease.

[30] Kim B, Feldman EL (2012). Insulin resistance in the nervous system. Trends in endocrinology and metabolism: TEM.

[31] Engel T, Lucas JJ, Hernandez F, Avila J (2007). A mouse model to study tau pathology related with tau phosphorylation and assembly. Journal of the neurological sciences.

[32] Hwang JJ, Park MH, Choi SY, Koh JY (2005). Activation of the Trk signaling pathway by extracellular zinc. Role of metalloproteinases. The Journal of biological chemistry.

[33] Bathina S, Das UN (2015). Brain-derived neurotrophic factor and its clinical implications. Archives of medical science: AMS.

[34] Sabio G (2008). A stress signaling pathway in adipose tissue regulates hepatic insulin resistance. Science.

[35] Bomfim TR (2012). An anti-diabetes agent protects the mouse brain from defective insulin signaling caused by Alzheimer’s disease- associated Abeta oligomers. The Journal of clinical investigation.

[36] Chua LM (2012). Impaired neuronal insulin signaling precedes Abeta42 accumulation in female AbetaPPsw/PS1DeltaE9 mice. Journal of Alzheimer’s disease: JAD.

[37] Ma QL (2009). Beta-amyloid oligomers induce phosphorylation of tau and inactivation of insulin receptor substrate via c-Jun N-terminal kinase signaling: suppression by omega-3 fatty acids and curcumin. The Journal of neuroscience: the official journal of the Society for Neuroscience.

[38] Reifert J, Hartung-Cranston D, Feinstein SC (2011). Amyloid beta-mediated cell death of cultured hippocampal neurons reveals extensive Tau fragmentation without increased full-length tau phosphorylation. The Journal of biological chemistry.

[39] Yamada M (1997). Insulin receptor substrate (IRS)-1 and IRS-2 are tyrosine-phosphorylated and associated with phosphatidylinositol 3-kinase in response to brain-derived neurotrophic factor in cultured cerebral cortical neurons. The Journal of biological chemistry.

[40] Zheng WH, Quirion R (2004). Comparative signaling pathways of insulin-like growth factor-1 and brain-derived neurotrophic factor in hippocampal neurons and the role of the PI3 kinase pathway in cell survival. Journal of neurochemistry.

[41] Mizoguchi H (2011). Matrix metalloproteinase-9 contributes to kindled seizure development in pentylenetetrazole-treated mice by converting pro-BDNF to mature BDNF in the hippocampus. The Journal of neuroscience: the official journal of the Society for Neuroscience.

[42] Kimura R, Devi L, Ohno M (2010). Partial reduction of BACE1 improves synaptic plasticity, recent and remote memories in Alzheimer’s disease transgenic mice. Journal of neurochemistry.

[43] Hirosumi J (2002). A central role for JNK in obesity and insulin resistance. Nature.

[44] Ebenezer PJ (2010). Neuron specific toxicity of oligomeric amyloid-beta: role for JUN-kinase and oxidative stress. Journal of Alzheimer’s disease: JAD.

[45] Zhu X (2001). Activation and redistribution of c-jun N-terminal kinase/stress activated protein kinase in degenerating neurons in Alzheimer’s disease. Journal of neurochemistry.

[46] Killick R (2014). Clusterin regulates beta-amyloid toxicity via Dickkopf-1-driven induction of the wnt-PCP-JNK pathway. Molecular psychiatry.

[47] Savage MJ, Lin YG, Ciallella JR, Flood DG, Scott RW (2002). Activation of c-Jun N-terminal kinase and p38 in an Alzheimer’s disease model is associated with amyloid deposition. The Journal of neuroscience: the official journal of the Society for Neuroscience.

[48] Oakley H (2006). Intraneuronal beta-amyloid aggregates, neurodegeneration, and neuron loss in transgenic mice with five familial Alzheimer’s disease mutations: potential factors in amyloid plaque formation. The Journal of neuroscience: the official journal of the Society for Neuroscience.

[49] Beaudoin GM (2012). Culturing pyramidal neurons from the early postnatal mouse hippocampus and cortex. Nature protocols.

[50] Nunez, J. Primary Culture of Hippocampal Neurons from P0 Newborn Rats. *Journal of visualized experiments: JoVE* (2008). 10.3791/895 PMC2872976 19066540

