# Supplementary material for: Overexpression of matrix metalloproteinase-9 (MMP-9) rescues insulin-mediated impairment in the 5XFAD model of Alzheimer’s disease
Source: Sci Rep. 2017 Apr 6;7:683. doi: 10.1038/s41598-017-00794-5 (PMC5429641; doi:10.1038/s41598-017-00794-5)

Supplementary Information

Overexpression of matrix metalloproteinase-9 (MMP-9) rescues insulin-mediated impairment in the 5xFAD model of Alzheimer's disease

Archontia Kaminari, Nikolas Giannakas, Athina Tzinia* and Effie C. Tsilibary*

*Corresponding Authors:

Athina Tzinia, PhD

Institute of Biosciences and Applications
NCSR "Demokritos"
Agia Paraskevi, Athens 15310, Greece
E-mail: atzin@bio.demokritos.gr
Phone number: +0030 210 6503678
Fax number: +0030 210 6511767

Effie C. Tsilibary, MD, PhD

Institute of Biosciences and Applications
NCSR "Demokritos"
Agia Paraskevi, Athens 15310, Greece
E-mail: effie@bio.demokritos.gr
Phone number: +0030 210 6503678
Fax number: +0030 210 6511767

Full-length Western blot images

Figure 1 A

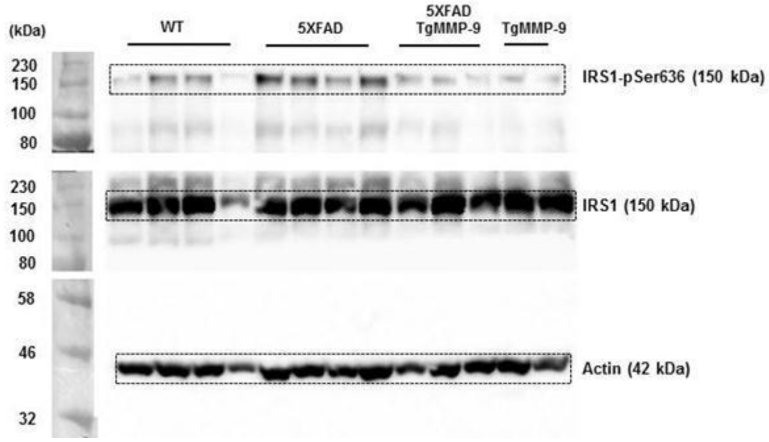

Figure 1 C

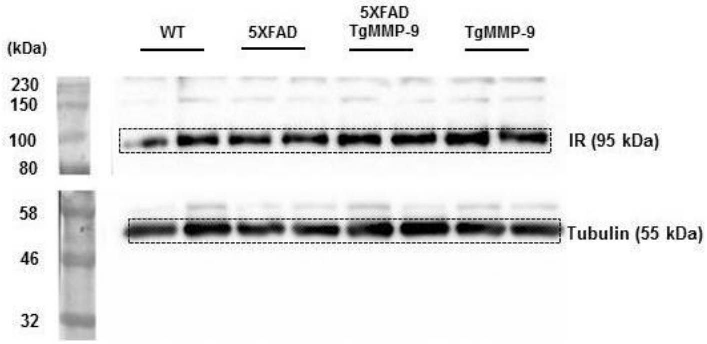

Figure 1 B, D

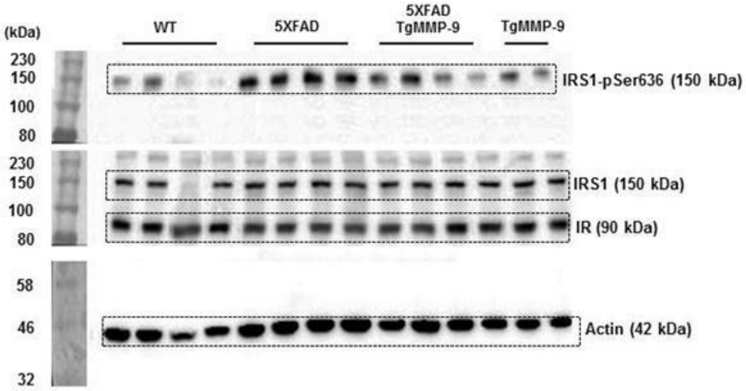

Figure 3 A, C

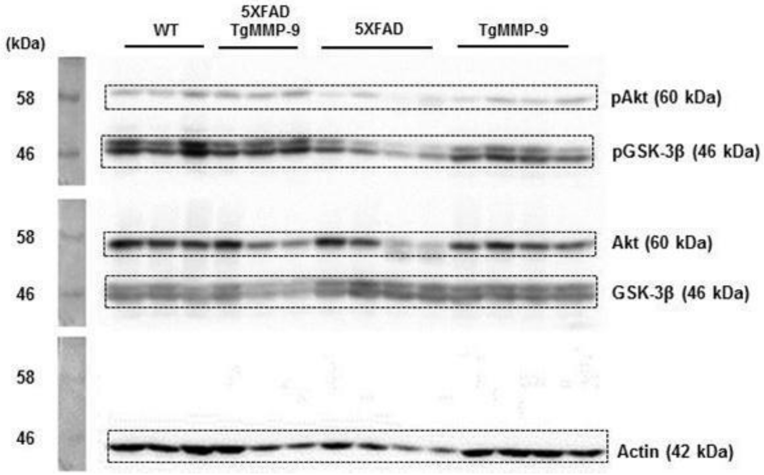

Figure 3 B

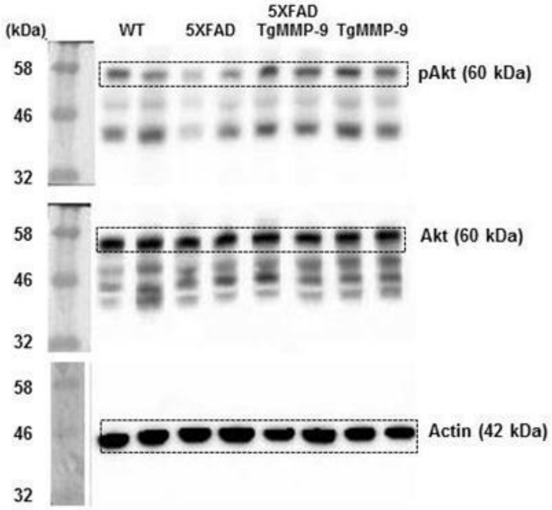

Figure 3 D

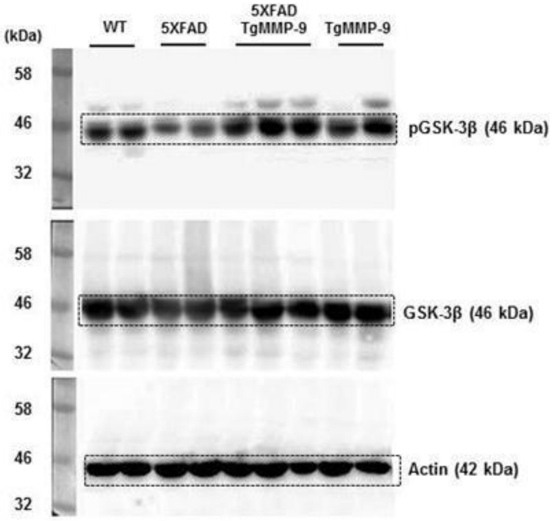

Figure 4 A

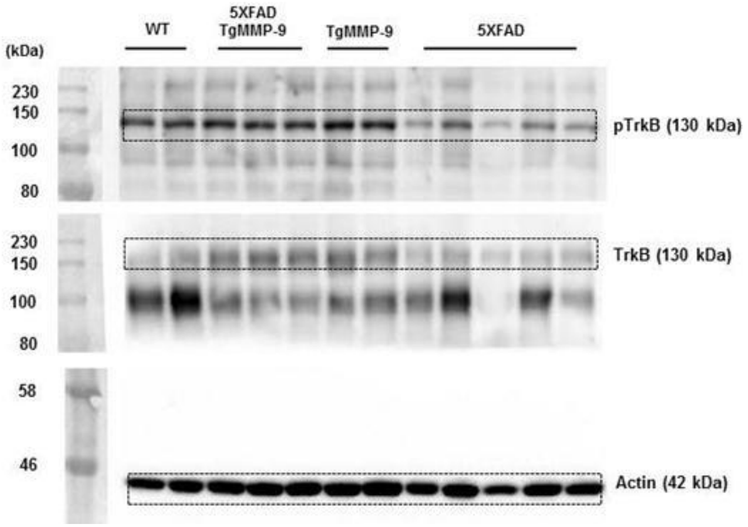

Figure 4 B

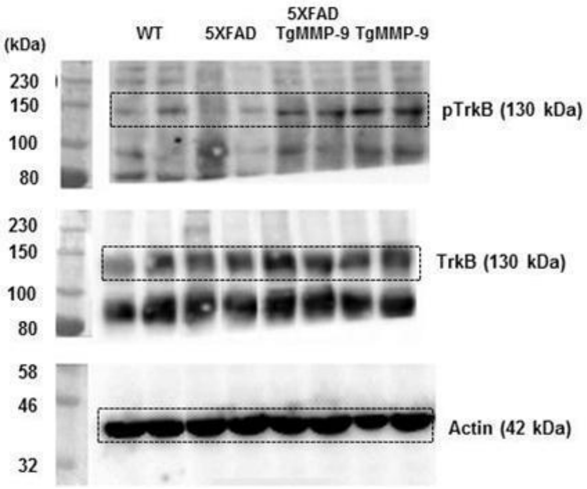

Figure 4 C

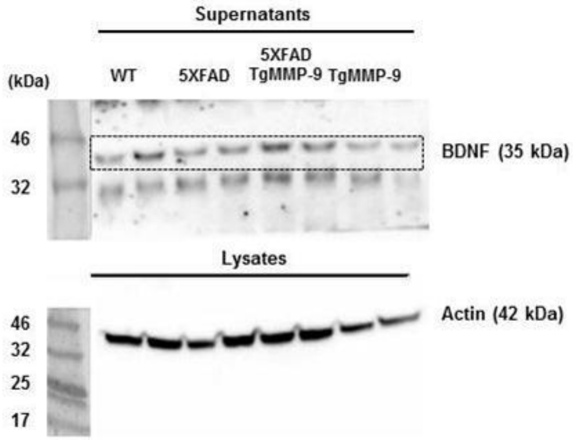

Figure 4 D

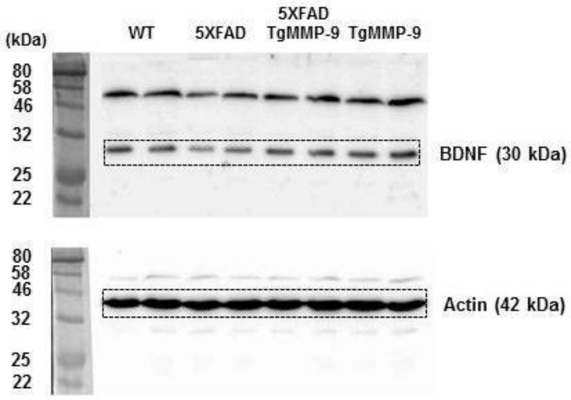

Figure 5 B

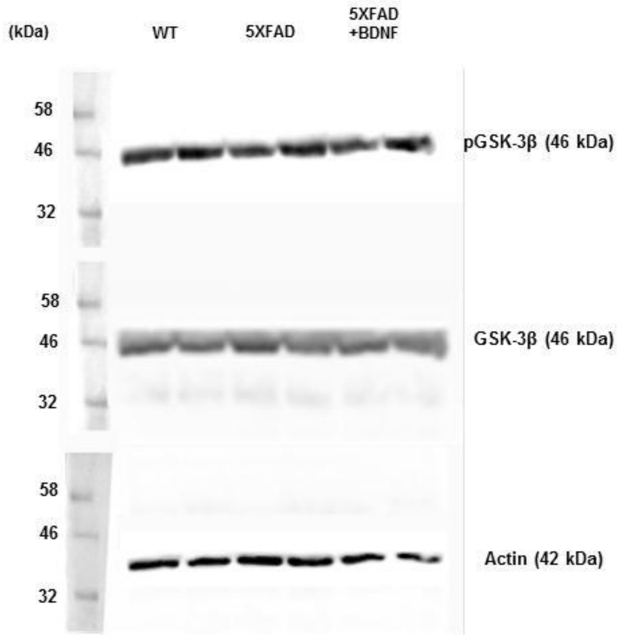

Figure 6 A

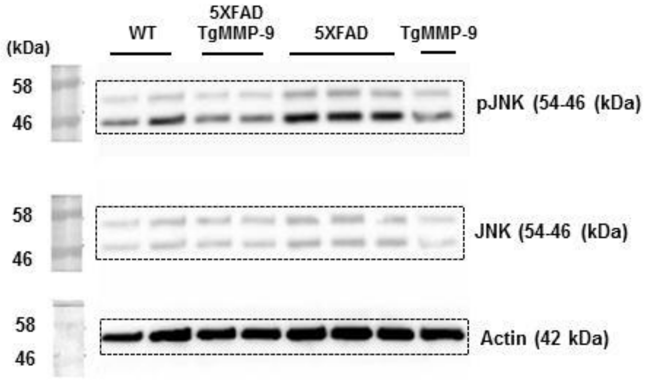

Figure 6 C

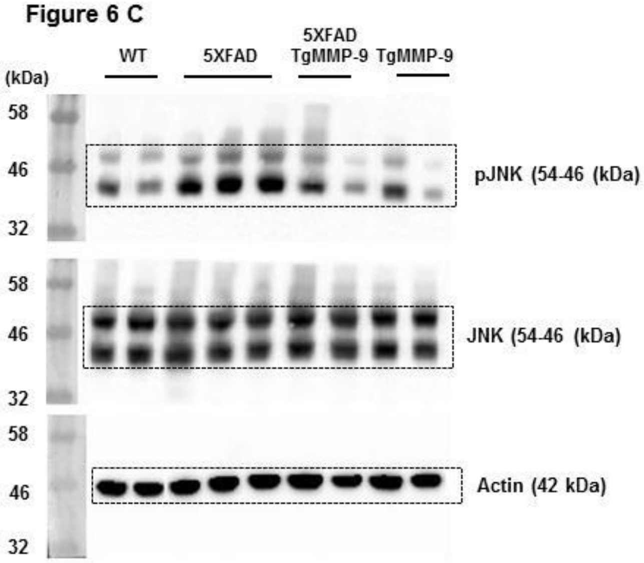

Figure 6 D

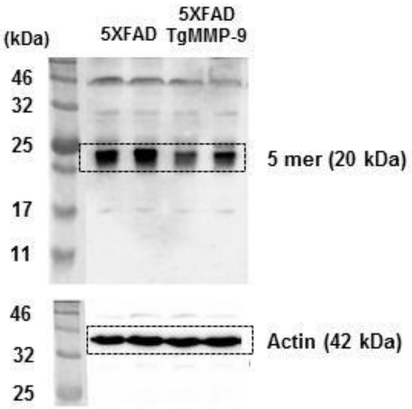

Supplement: Supplementary file 1 — Supplementary Information [file 41598_2017_794_MOESM1_ESM.pdf]
